# Stability analysis and exploration of multiform soliton solutions for extended fractional NLS model using modified extended direct algebraic method

**DOI:** 10.1038/s41598-026-48474-7

**Published:** 2026-05-06

**Authors:** Mahmoud Soliman, M. Elsaid Ramadan, Soliman Alkhatib, Hamdy M. Ahmed

**Affiliations:** 1 Department of Physics and Engineering Mathematics, Faculty of Engineering, Ain Shams University, Cairo, Egypt; 2 Department of Mathematics, Islamic University of Madinah, Medina, Saudi Arabia; 3 College of Engineering and Technology (CET), American University in the Emirates (AUE),Dubai intel Academic City, P.O. Box 503000, Dubai, United Arab Emirates; 4 Department of Physics and Engineering Mathematics, Higher Institute of Engineering, El Shorouk Academy, Cairo, Egypt

**Keywords:** Fractional derivative, Solitons, Jacobi elliptic waves, Nonlinear fractional system, Stability, Mathematics and computing, Optics and photonics, Physics

## Abstract

This paper investigates a generalized nonlinear Schrödinger-type equation involving fractional derivatives in both space and time, formulated through the recently introduced $\beta$-fractional operator. The modified extended direct algebraic method (MEDM) is applied to derive new classes of exact analytical solutions, including bright, dark, periodic, and singular solitons. A detailed analysis of the fractional parameters reveals their quantitative influence on soliton width, velocity, and localization. The modulation instability (MI) gain spectra are evaluated to identify stability regions and illustrate how decreasing fractional orders suppress instability growth. Physically, the results demonstrate that the fractional orders act as tunable parameters governing wave dispersion, nonlocality, and energy localization in nonlinear media. The study establishes a flexible framework for controlling soliton dynamics in optical and plasma systems, underscoring the fundamental role of fractional calculus in modeling complex nonlinear wave phenomena.

## Introduction

Nonlinear partial differential equations (NLPEs) form the mathematical bedrock for describing a vast array of complex physical phenomena observed across engineering, physics, and biology. From the macro-scale dynamics of fluid flow and cosmology to the micro-scale interactions governing plasma physics and nonlinear optics, NLPEs are essential tools for modeling systems where multiple competing physical effects dictate overall behavior^1–9^. Consequently, determining the exact analytic solutions of these equations is not merely an academic exercise, but a critical necessity. Such exact solutions provide deep, fundamental insight into the underlying mechanisms of wave propagation, stability, and energy transport, often illuminating physical constraints and scaling laws that approximate or numerical methods might obscure. The quest for robust and versatile solution methods has driven significant advancements in computational and analytical mathematics, yielding techniques such as the extended Bernoulli’s equation approach^10^, the Jacobi elliptic function expansion, the Kudryashov method^11^, the Hirota bilinear formalism^12,13^, and the F-expansion technique^14^.

One of the most widely applied NLPEs is the Nonlinear Schrödinger (NLS) equation, which is fundamental to understanding wave dynamics in dispersive and nonlinear media, especially within the field of optical fiber communications. The classical NLS model successfully describes the basic propagation of optical pulses by balancing Group Velocity Dispersion (GVD) and the Kerr nonlinearity^15–19^.

However, modern, high-capacity, and ultra-fast fiber optic networks face dynamics that quickly exceed the capability of the standard NLS equation. This reality necessitates the use of extended NLS models that incorporate higher-order effects. These extensions account for critical phenomena like third- and fourth-order dispersion, self-steepening, and stimulated scattering effects, which become pronounced when dealing with ultra-short pulses (femtosecond scale). Several notable models have emerged to capture these complexities, including the Biswas-Arshed model^20,21^, the Fokas-Lenells equation^22,23^ (derived via inverse scattering theory), the Chen-Lee-Liu equation^24^, and the powerful Radhakrishnan-Kundu-Lakshmanan (RKL) equation^25,26^. The rigorous analysis of these high-order models is central to designing effective strategies for dispersion management, which ultimately dictates the performance and bandwidth of modern telecommunication infrastructure.

The present study adopts a fractional nonlinear Schrödinger-type equation formulated with the recently introduced $\beta$-fractional derivative to describe nonlinear dispersive wave. The main motivation for selecting this model lies in its ability to capture anomalous transport and hereditary effects that are not represented in the classical integer-order NLS framework. Conventional models assume local dispersion and instantaneous nonlinear response, which limit their applicability to complex optical^27,28^ or plasma systems^29,30^ exhibiting long-range correlations. Although various fractional derivatives have been employed in nonlinear dynamics, previous works have primarily considered Caputo or Riemann–Liouville operators, neglecting the combined spatial–temporal effects that the $\beta$-derivative can represent. Moreover, the influence of dual fractional parameters $(\alpha ,\beta )$ on soliton width, stability, and localization remains largely unexplored. This study aims to fill this gap by developing and analyzing exact analytical solutions of the fractional NLS model using the modified extended direct algebraic method (MEDM) and by establishing the physical connection between fractional parameters and the tunable control of nonlinear wave behavior.

This paper specifically addresses the complex, extended fractional $\beta$-derivative Schrödinger equation, as previously described in the literature^31^:

1 $$\begin{aligned} & \frac{D^{\alpha }{}_{\text {xx}}u}{2}+i D_t^{\beta }u+u|u|^2-i \sigma \left( D^{\alpha }{}_{\text {xxx}}u+6 D^{\alpha }_xu~|u|^2\right) \nonumber \\ & + \gamma \left( D^{\alpha }{}_{\text {xxxx}}u+6 u^*\left| D^{\alpha }{}_x u|^2+4 u\right| D^{\alpha }{}_x u|^2+8 \left( D^{\alpha }{}_{\text {xx}}u\right) {}^*|u|^2+2 u^2 \left( D^{\alpha }{}_{\text {xx}} u\right) {}^*+6 u|u|^4\right) =0.\end{aligned}$$

In this equation, the dependent variable *u*(*x*, *t*) represents the complex-valued optical field. The terms $D^{\alpha }_{\text {xx}}u$ and $D_t^{\beta }u$ represent dispersion and temporal evolution, respectively, governed by the fractional orders $\alpha$ (space) and $\beta$ (time), where $0 < \alpha , \beta \le 1$. The term $u|u|^2$ represents the Kerr nonlinearity, while $\sigma$ and $\gamma$ scale the various high-order dispersive ($\alpha$-order derivatives) and nonlinear terms.

Previous studies have demonstrated that fractional Schrödinger equations play a fundamental role in modeling pulse propagation, revealing that fractional-order parameters have a pronounced influence on soliton stability and dynamical behavior^32–34^. However, the analytical treatment of Equation (1) remains highly challenging due to its high-order structure, intrinsic nonlinearity, and the presence of coupled space–time fractional derivatives, which necessitate an efficient and powerful solution framework.

To address these challenges, the present work adopts the Modified Direct Algebraic Method (MEDM). This method is particularly effective for dealing with high-order derivatives and multi-term nonlinearities inherent in fractional models. By applying MEDM, a broad class of exact wave solutions is derived, encompassing elementary structures such as exponential-type solutions and bright solitons, as well as more sophisticated periodic waveforms expressed in terms of Jacobi elliptic and Weierstrass elliptic functions.

The remainder of this paper is organized as follows. “Preliminaries” section outlines the fundamental concepts of fractional calculus. “The Modified extended direct algebraic method (MEDM)” section provides a clear and concise description of the MEDM for solving fractional differential equations. In “Application to the Proposed Model” section, this methodology is systematically applied to Equation (1), and the newly obtained exact solutions are presented. “Graphical Simulations of Selected Solutions” section illustrates the physical behavior of these solutions through graphical simulations, highlighting the significant influence of the fractional parameters $\alpha$ and $\beta$ on wave propagation. Finally, “Linear stability analysis” section concludes the paper and discusses possible directions for future research.

## Preliminaries

In this section, we provide basic mathematical preliminaries for the fractional derivative used in this paper.

The $\beta -$fractional derivative is a local fractional operator. It modifies the classical derivative preserving the basic structure of integer-order calculus (linearity, product rule, chain rule) without involving integral kernels or historical memory integrals.

The $\beta$-fractional derivative is defined as^35–37^:

2 $$\begin{aligned} D_t^{\beta }v(t) = \lim _{h \rightarrow 0} \frac{v\!\left( t + h\left( t + \frac{1}{\Gamma (\beta )}\right) ^{1-\beta }\right) - v(t)}{h},~t>0,~ 0 < \beta \le 1. \end{aligned}$$

This operator modifies the temporal scaling of the governing equation while preserving the local structure of the differential operator. Unlike classical nonlocal fractional derivatives, it does not introduce history dependence, but instead provides additional flexibility in describing anomalous temporal evolution in nonlinear optical and quantum mechanical systems.

### Properties of the $\beta$-fractional derivative

The $\beta$-derivative retains several essential properties analogous to those of the classical derivative, which are listed below for any scalar constants $a, b \in \mathbb {R}$ and differentiable functions $f(t)$, $g(t)$:

- *Linearity*
  $$\begin{aligned} D_t^{\beta }\!\big [a f(t) + b g(t)\big ] = a D_t^{\beta } f(t) + b D_t^{\beta } g(t). \end{aligned}$$
- *Product Rule*
  $$\begin{aligned} D_t^{\beta }\!\big [f(t)g(t)\big ] = f(t) D_t^{\beta } g(t) + g(t) D_t^{\beta } f(t). \end{aligned}$$
- *Quotient Rule*
  $$\begin{aligned} D_t^{\beta }\!\left[ \frac{f(t)}{g(t)}\right] =\frac{ g(t) D_t^{\beta } f(t) - f(t) D_t^{\beta } g(t) }{[g(t)]^2}, \qquad g(t) \ne 0. \end{aligned}$$
- *Derivative of a Constant*
  $$\begin{aligned} D_t^{\beta }[c] = 0, \qquad c \in \mathbb {R}. \end{aligned}$$
- *Power Function*
  $$\begin{aligned} D_t^{\beta }\!\big [t^{n}\big ] = \frac{\Gamma (n+1)}{\Gamma (n+1-\beta )}\, t^{n-\beta }, \qquad n > -1. \end{aligned}$$

These properties simplify algebraic manipulations and enable analytical derivations of fractional differential equations, such as those in the extended NLS framework.

#### Physical interpretation and limiting case

The fractional order $\beta$acts as a control parameter that regulates the temporal scaling of the system dynamics and influences the rate of wave evolution. For $\beta \rightarrow 1$, Eq. (3) smoothly reduces to the standard first-order derivative:

$$\begin{aligned} \lim _{\beta \rightarrow 1} D_t^{\beta }v(t) = \frac{dv}{dt}, \end{aligned}$$

recovering the classical nonlinear Schrödinger model. For $0< \beta < 1$, the fractional derivative introduces subdiffusive dynamics and long-range correlations, allowing the modeling of processes with anomalous transport or delay.

#### Comparison with other fractional derivatives

Several operators have been proposed to define fractional differentiation. The most widely used are summarized as follows:

- *Caputo Derivative* Incorporates initial conditions in the same form as classical calculus, making it suitable for physical boundary-value problems.
- *Riemann--Liouville Derivative:* Useful for theoretical derivations, though it yields non-zero derivatives for constants, complicating physical interpretation.
- *Atangana--Baleanu Derivative* Defined using a non-singular Mittag–Leffler kernel, which provides smooth memory decay and enhanced numerical stability.

Compared to these, the $\beta$-fractional derivative is a local operator that extends the classical derivative while preserving many of its fundamental properties, making it particularly attractive for analytical studies. It satisfies the standard rules of calculus, and it naturally reduces to the classical derivative as the fractional order approaches unity. Unlike nonlocal fractional derivatives, the $\beta$-derivative avoids singular kernel integrals and long-range memory effects, which significantly simplifies both the mathematical formulation and solution process. As a result, it is more tractable and well suited for analytical techniques such as the Modified Extended Direct Algebraic Method, enabling the efficient derivation of exact traveling wave and soliton solutions. From a physical perspective, the fractional $\beta$-derivative effectively captures local scaling and deformation phenomena, allowing the fractional parameters to serve as tuning controls for wave propagation which local fractional effects dominate and long-memory behavior is not required.

## The modified extended direct algebraic method (MEDM)

This section details the analytical framework of the modified direct algebraic Method (MEDM)^38^, which is used to derive exact traveling wave solutions for the studied fractional nonlinear Schrödinger equation.

We begin by considering a general nonlinear partial differential equation (NLPDE) involving fractional derivatives:

3 $$\begin{aligned} V(u, u_t, u_x, u_{xx}, u_{tx}, \dots ) = 0. \end{aligned}$$

The application of the MEDM involves a systematic sequence of steps to reduce this fractional NLPDE into a solvable algebraic system.

### Methodological steps

#### Step 1: traveling wave transformation

The core of the method is transforming the NLPDE into a single ordinary differential equation (ODE). We apply the wave transformation using a new wave profile function $Q(\xi )$ and phase function $\Phi$, where $\xi$ is the traveling wave variable:

4 $$\begin{aligned} u(x,t)=Q(\xi ) ~e^{i\Phi }, \end{aligned}$$

where the wave variable $\xi$ and phase $\Phi$ are defined by:

$$\begin{aligned} \xi =\frac{c_1 \left( \frac{1}{\Gamma (\alpha )}+x\right) ^{\alpha }}{\alpha }-\frac{c_2 \left( \frac{1}{\Gamma (\beta )}+t\right) ^{\beta }}{\beta }, \quad \Phi =\theta -\frac{c_3 \left( \frac{1}{\Gamma (\alpha )}+x\right) ^{\alpha }}{\alpha }+\frac{c_4 \left( \frac{1}{\Gamma (\beta )}+t\right) ^{\beta }}{\beta }. \end{aligned}$$

Here, $\alpha$ and $\beta$ are the fractional orders, $c_1$ and $c_2$ determine the spatial and temporal parameters of the wave profile, and $c_3$ and $c_4$ represent the wave number and frequency, respectively.

It is important to note that the similarity transformation is formulated in its most general form in order to accommodate independent spatial and temporal fractional orders. In the present model, the parameter $\alpha$ governs the spatial fractional dispersion, while $\beta$ characterizes the temporal fractional evolution associated with the $\beta$-fractional derivative operator. Treating these parameters independently enables the model to capture distinct anomalous transport mechanisms in space and time, which are frequently observed in complex nonlinear media such as optical fibers, plasmas, and viscoelastic fluids. For simplicity, a reduced formulation can be obtained by setting $\alpha = \beta$, which leads to a unified fractional scaling for both spatial and temporal variables. In this case, the similarity transformation retains the same analytical structure, and the subsequent solution procedure remains unchanged. Therefore, the transformation introduced above represents the most general framework, while the single-order scenario may be considered as a special limiting case of the present model. Substituting these transformations into Equation (3) yields the complex ODE, which is then separated into its real and imaginary parts to obtain a single real-valued ODE for $Q(\xi )$:

5 $$\begin{aligned} {\mathcal {H}}(Q, Q', Q'', Q''', Q^{(4)}, \dots ) = 0. \end{aligned}$$

#### Step 2: postulated solution form

The solution for the resulting ODE, $Q(\xi )$, is assumed to be represented by a finite Laurent series in terms of an internal auxiliary function $\Phi (\xi )$:

6 $$\begin{aligned} Q(\xi )=\sum _{j=0}^{N}{a_j \Phi ^j(\xi )}+\sum _{j=-1}^{-N}{b_{-j} \Phi ^j(\xi )}, \end{aligned}$$

where the auxiliary function $\Phi (\xi )$ satisfies the following first-order nonlinear ordinary differential equation:

7 $$\begin{aligned} \Phi '(\xi ) = \sqrt{d_0 + d_1 \Phi (\xi ) + d_2 \Phi ^2(\xi ) + d_3 \Phi ^3(\xi )+ d_4 \Phi ^4(\xi ) + d_6 \Phi ^6(\xi )}. \end{aligned}$$

where $d_0, d_1, d_2, d_3, d_4, d_6$ are constants coefficients.

#### Step 3: determination of the truncation order *N*

The truncation order *N* is determined by equating the highest derivative term in Equation (5) with the highest power of the nonlinear term in $Q(\xi )$, following the principle of homogeneous balancing.

#### Step 4 & 5: algebraic system derivation and solution

We substitute the proposed solution form (Eq. (6)) and the auxiliary equation (Eq. (7)) into the ODE (Eq. (5)). By gathering the coefficients of $\Phi ^p(\xi )$ and setting each coefficient to zero, we establish a system of nonlinear algebraic equations. This system is solved using computational software (e.g., Mathematica) to find the unknown parameters $a_j$, $b_j$, and the traveling wave coefficients $c_1, c_2, c_3, c_4$.

#### Step 6: auxiliary equation solutions (special cases)

Various analytical forms for the function $\Phi (\xi )$ can be derived by selecting specific values for the parameters $d_0$ through $d_4$. These cases include:

*Case 1: Soliton Solutions Setting* $d_0=d_1=d_3=d_6=0$, the solution yields hyperbolic functions corresponding to soliton solutions:

$$\begin{aligned} \Phi (\xi )=\sqrt{-\frac{d_2}{d_4}} \text {sech}(\sqrt{d_2} \xi ),\quad d_2>0, d_4<0. \end{aligned}$$

*Case 2: Jacobi Elliptic Solutions* Setting $d_1=d_3=d_6=0$ leads to solutions expressed in terms of Jacobi elliptic functions, which represent periodic wave structures:

$$\begin{aligned} \Phi (\xi )=\sqrt{\frac{-d_2 m^{2}}{d_4(2m^{2}-1)}} \text {cn}\left(\sqrt{\frac{d_2}{(2m^{2}-1)}} \xi \right),\quad d_2>0, d_4<0 , d_0=\frac{d_2^{2} m^{2}(1-m^{2})}{d_4 (2m^{2}-1)^{2}}, \end{aligned}$$

and

$$\begin{aligned} \Phi (\xi )=\sqrt{-\frac{m^{2}}{d_4(2-m^{2})}} \text {dn}\left(\sqrt{\frac{d_2}{(2-m^{2})}} \xi \right),\quad d_2>0, d_4<0 , d_0=\frac{d_2^{2} (1-m^{2})}{d_4 (2-m^{2})^{2}}, \end{aligned}$$

where *m* is the elliptic modulus ($0 < m \le 1$).

*Case 3: Weierstrass Elliptic Solutions* Setting $d_{2}=d_{4}=d_6=0$ leads to solutions in terms of the Weierstrass elliptic function ($\wp$):

$$\begin{aligned} \Phi (\xi )=\wp \left(\frac{\sqrt{d_{3}}}{2}\xi ,\dfrac{-4d_{1}}{d_{3}},\dfrac{-4d_{0}}{d_{3}}\right). \end{aligned}$$

*Case 4: Exponential Solutions* Setting $d_{0}=d_{1}=d_6=d_{2}=0$ results in simple exponential function solutions:

$$\begin{aligned} \Phi (\xi )=\frac{d_3}{2d_4} \exp \left(\frac{d_3}{2\sqrt{-d_4}}\xi \right). \end{aligned}$$

#### Step 7: final solution construction

The final exact solution for the nonlinear fractional equation (Eq. (3)) is constructed by substituting the derived coefficients $a_j, b_{-j}$ and the corresponding specific auxiliary function $\Phi (\xi )$ (from Step 6) back into the main solution form $u(x,t)=Q(\xi )e^{i\Phi }$.

## Application to the proposed model

In this section, we construct exact analytical solutions of Eq. (1) by employing the transformation

8 $$\begin{aligned} u(x,t) = Q(z)\, e^{i\phi }, \quad z = \frac{h}{\alpha }\left( \frac{1}{\Gamma (\alpha )}+x\right) ^{\alpha } - \frac{\nu }{\beta }\left( \frac{1}{\Gamma (\beta )}+t\right) ^{\beta }, \quad \phi = \theta - \frac{k}{\alpha }\left( \frac{1}{\Gamma (\alpha )}+x\right) ^{\alpha } + \frac{\omega }{\beta }\left( \frac{1}{\Gamma (\beta )}+t\right) ^{\beta }. \end{aligned}$$

Here, $h$ and $\nu$ denote the spatial and temporal scaling parameters of the wave variable, while $k$ and $\omega$ represent the wave number and frequency, respectively. The constants $\sigma$ and $\gamma$ quantify higher-order dispersion and nonlinear effects.The transformations introduced in Eq. (8) represent a similarity reduction that couples the spatial and temporal coordinates into a single self-similar variable. Physically, this corresponds to moving into the co-propagating frame of the localized wave packet, where the balance between nonlinear and dispersive effects is most naturally described. Such transformations convert the governing fractional partial differential equation into an ordinary differential form, enabling the extraction of analytical soliton solutions and revealing the propagation invariance of the underlying wave structure. Similar travelling-wave transformations have been widely employed in studies of fractional and nonlinear Schrödinger-type equations ^39,40^.

The transformation introduced in Eq. (9) is formulated for the general space--time fractional model, where the spatial and temporal derivatives may possess different fractional orders $\alpha$ and $\beta$. In this framework, $\alpha$ characterizes the spatial fractional dispersion, while $\beta$ governs the temporal fractional evolution of the wave field. Keeping these parameters independent enables the model to describe distinct anomalous scaling behaviors in space and time, which frequently arise in nonlinear dispersive systems. For simplicity, the reduced case $\alpha = \beta$ may also be considered. Under this condition, the transformation and the analytical solutions presented below remain valid and naturally reduce to a single-order fractional formulation without altering the structure of the obtained solutions. Substituting Eq. (8) into Eq. (1) transforms the original fractional nonlinear PDE into a complex ordinary differential equation. By separating the resulting expression into its real and imaginary components, we obtain the following system:

9 $$\begin{aligned} & 2\gamma h^{4} Q^{(4)}(z) +h^{2}(-12\gamma k^{2}-6k\sigma +1)Q''(z) +Q(z)\left( 20\gamma h^{2}Q'(z)^{2}+2\gamma k^{4}+2k^{3}\sigma -k^{2}-2\omega \right) \nonumber \\ & +20\gamma h^{2}Q(z)^{2}Q''(z) -2Q(z)^{3}(12\gamma k^{2}+6k\sigma -1) +12\gamma Q(z)^{5}=0, \end{aligned}$$

10 $$\begin{aligned} 2Q'(z)\!\left[ h k(4\gamma k^{2}+3k\sigma -1)-6hQ(z)^{2}(4\gamma k+\sigma )-\nu \right] -2h^{3}(4\gamma k+\sigma )\, Q^{(3)}(z)=0. \end{aligned}$$

Balancing the coefficients resulting from Eq. (12) gives

11 $$\begin{aligned} k = -\frac{\sigma }{4\gamma }, \qquad \nu = h k\left( 4\gamma k^{2}+3k\sigma -1\right) . \end{aligned}$$

To proceed with the MEDM methodology, the integer $N$ must be identified. Balancing the highest derivative term $Q^{(4)}$ with the nonlinear contribution $Q'' Q^{2}$ yields:

$$\begin{aligned} N=1. \end{aligned}$$

Thus, the solution of the reduced ODE can be written as

12 $$\begin{aligned} Q(z) = s_{0} + s_{1}\Upsilon (z) + \frac{r_{1}}{\Upsilon (z)}. \end{aligned}$$

Substituting Eqs. (12) and (8) into Eq. (9), and equating the coefficients of similar powers of $\Upsilon (z)$, produces a nonlinear algebraic system. This system is solved using Mathematica, yielding the exact solutions of Eq. (1).

**Case 1:** $$d_{0}=d_6=d_{1}=d_{3}=0$$

$$\begin{aligned} & s_{0}=0,\\ & s_{1}=\sqrt{-d_{4}}\,h,\\ & s_{2}=0,\\ & \omega =\frac{-8\gamma \sigma ^{2}+256\gamma ^{4}d_{2}^{2}h^{4}+128\gamma ^{3}d_{2}h^{2} +96\gamma ^{2}d_{2}h^{2}\sigma ^{2}-3\sigma ^{4}}{256\gamma ^{3}}. \end{aligned}$$

This leads to a bright soliton solution:

13 $$\begin{aligned} \begin{aligned} u(x,t)&= h\sqrt{d_{2}}\, \textrm{sech}\!\left[ \sqrt{d_{2}} \left( \frac{h\sigma \left( -\frac{\sigma ^{2}}{2\gamma }-1\right) \left( \frac{1}{\Gamma (\beta )}+t\right) ^{\beta }}{4\beta \gamma } +\frac{h\left( \frac{1}{\Gamma (\alpha )}+x\right) ^{\alpha }}{\alpha } \right) \right] \\&\quad \times \exp \!\left[ i\left( \theta - \frac{k\left( \frac{1}{\Gamma (\alpha )}+x\right) ^{\alpha }}{\alpha } + \frac{\omega \left( \frac{1}{\Gamma (\beta )}+t\right) ^{\beta }}{\beta } \right) \right] . \end{aligned} \end{aligned}$$

**Case 2:** $$d_{1}=d_6=d_{3}=0,\quad d_{0}=\dfrac{d_{2}^{2}P}{d_{4}}$$

$$\begin{aligned} & s_{0}=0,\\ & s_{1}=\sqrt{-d_{4}}\,h,\\ & s_{2}=0,\\ & \omega =\frac{-8\gamma \sigma ^{2}+256\gamma ^{4}d_{2}^{2}h^{4} +512\gamma ^{4}d_{2}^{2}h^{4}P+128\gamma ^{3}d_{2}h^{2} +96\gamma ^{2}d_{2}h^{2}\sigma ^{2}-3\sigma ^{4}}{256\gamma ^{3}}. \end{aligned}$$

Corresponding Jacobi elliptic-type waveforms are obtained:

14 $$\begin{aligned} \begin{aligned} u(x,t)&=h\sqrt{\frac{m^{2}}{2-m^{2}}}\, \textrm{dn}\!\left[ \left. \left( \frac{h\left( x+\frac{1}{\Gamma (\alpha )}\right) ^{\alpha }}{\alpha } -\frac{\nu \left( t+\frac{1}{\Gamma (\beta )}\right) ^{\beta }}{\beta } \right) \sqrt{\frac{d_{2}}{2-m^{2}}} \right| m\right] \\&\quad \times \exp \!\left[ i\left( \theta - \frac{k\left( \frac{1}{\Gamma (\alpha )}+x\right) ^{\alpha }}{\alpha } + \frac{\omega \left( \frac{1}{\Gamma (\beta )}+t\right) ^{\beta }}{\beta }\right) \right] . \end{aligned} \end{aligned}$$

15 $$\begin{aligned} \begin{aligned} u(x,t)&=h\sqrt{\frac{d_{2}m^{2}}{2m^{2}-1}}\, \textrm{cn}\!\left[ \left. \left( \frac{h\left( x+\frac{1}{\Gamma (\alpha )}\right) ^{\alpha }}{\alpha } -\frac{\nu \left( t+\frac{1}{\Gamma (\beta )}\right) ^{\beta }}{\beta } \right) \sqrt{\frac{d_{2}}{2m^{2}-1}} \right| m\right] \\&\quad \times \exp \!\left[ i\left( \theta - \frac{k\left( \frac{1}{\Gamma (\alpha )}+x\right) ^{\alpha }}{\alpha } + \frac{\omega \left( \frac{1}{\Gamma (\beta )}+t\right) ^{\beta }}{\beta }\right) \right] . \end{aligned} \end{aligned}$$

**Case 3.** For the parameter choice $d_{2}=d_6=d_{4}=0$ with $d_{3}>0$, the algebraic system yields

$$\begin{aligned} & s_{1}=0,\\ & s_{2}=\sqrt{-d_{0}}\,h,\\ & \omega =\frac{-8\gamma \sigma ^{2}-3\sigma ^{4} +11776\gamma ^{4}s_{0}^{4}+768\gamma ^{3}s_{0}^{2} +576\gamma ^{2}s_{0}^{2}\sigma ^{2}}{256\gamma ^{3}},\\ & d_{1}=-\frac{4\sqrt{-d_{0}}\,s_{0}}{h},\\ & d_{3}=\frac{8s_{0}^{3}}{\sqrt{-d_{0}}\,h^{3}}. \end{aligned}$$

This configuration leads to a Weierstrass elliptic-type solution of the form

16 $$\begin{aligned} u(x,t)= & \frac{\sqrt{-d_{0}}\,h}{ \wp \!\left( \sqrt{2} \left( \frac{h\left( x+\frac{1}{\Gamma (\alpha )}\right) ^{\alpha }}{\alpha } -\frac{\nu \left( t+\frac{1}{\Gamma (\beta )}\right) ^{\beta }}{\beta } \right) \sqrt{\frac{s_{0}^{3}}{h^{3}\sqrt{-d_{0}}}};\,-\frac{2h^{2}d_{0}}{s_{0}^{2}},-\frac{h^{3}\sqrt{-d_{0}}\,d_{0}}{2s_{0}^{3}} \right) } +s_{0} \nonumber \\ & \times \exp \!\left[ i\left( \theta -\frac{k\left( \frac{1}{\Gamma (\alpha )}+x\right) ^{\alpha }}{\alpha } +\frac{\omega \left( \frac{1}{\Gamma (\beta )}+t\right) ^{\beta }}{\beta } \right) \right] . \end{aligned}$$

**Case 4.** Let $d_{3}=d_6=d_{4}=0$ and $d_{0}=\dfrac{d_{1}^{2}}{4d_{2}}$. Solving the algebraic relations gives

$$\begin{aligned} & s_{0}=\frac{\sqrt{-4\gamma -3\sigma ^{2}}}{4\sqrt{3}\gamma },\\ & s_{1}=-\frac{\sqrt{3}\gamma \,d_{3}h^{2}}{\sqrt{-4\gamma -3\sigma ^{2}}},\\ & s_{2}=0,\\ & \omega =\frac{-32\gamma ^{2}-72\gamma \sigma ^{2}-27\sigma ^{4}}{768\gamma ^{3}},\\ & d_{4}=\frac{3\gamma ^{2}d_{3}^{2}h^{2}}{4\gamma +3\sigma ^{2}}. \end{aligned}$$

Under these parameter constraints, Eq. (1) admits the exponential-type solution

17 $$\begin{aligned} u(x,t)= & \left( \frac{\sqrt{-4\gamma -3\sigma ^{2}}}{4\sqrt{3}\gamma } -\frac{(4\gamma +3\sigma ^{2}) \exp \!\left[ \frac{ d_{3}\left( \frac{h\left( \frac{1}{\Gamma (\alpha )}+x\right) ^{\alpha }}{\alpha } -\frac{\nu \left( \frac{1}{\Gamma (\beta )}+t\right) ^{\beta }}{\beta } \right) }{ 2\sqrt{3}\sqrt{ -\frac{\gamma ^{2}d_{3}^{2}h^{2}}{4\gamma +3\sigma ^{2}} }} \right] }{ 2\sqrt{3}\gamma \sqrt{-4\gamma -3\sigma ^{2}} } \right) \nonumber \\ & \times \exp \!\left[ i\left( \theta -\frac{k\left( \frac{1}{\Gamma (\alpha )}+x\right) ^{\alpha }}{\alpha } +\frac{\omega \left( \frac{1}{\Gamma (\beta )}+t\right) ^{\beta }}{\beta } \right) \right] . \end{aligned}$$

## Graphical simulations of selected solutions

To highlight the behavior of the analytical solutions obtained in the previous section, several representative wave profiles are displayed through graphical simulations.

**Fig. 1:**
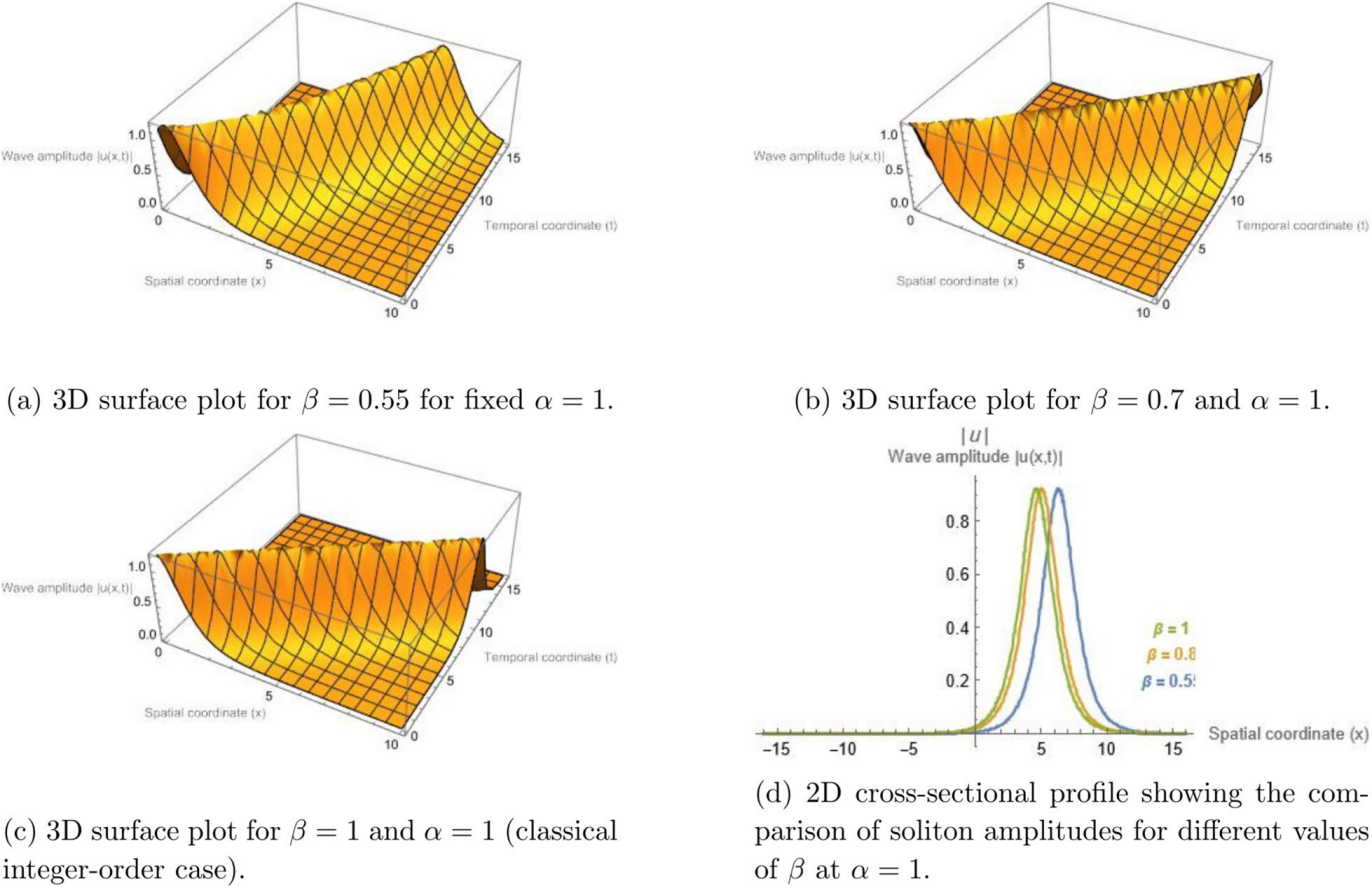
Bright soliton solution of Eq. (13) illustrating the influence of the temporal fractional order $\beta$ for a fixed spatial fractional order $\alpha = 1$. Panels (a)–(c) present three-dimensional surface plots of the wave amplitude |*u*(*x*, *t*)| for $\beta = 0.55$, $\beta = 0.7$, and $\beta = 1$, respectively. In these plots, the horizontal axis represents the spatial coordinate *x*, the depth axis corresponds to the temporal coordinate *t*, and the vertical axis denotes the wave amplitude |*u*(*x*, *t*)|. Panel (d) shows a two-dimensional cross-sectional comparison of the corresponding soliton envelopes for different values of $\beta$. The results illustrate how variations in the temporal fractional parameter influence the localization and propagation behavior of the soliton solution.

**Fig. 2:**
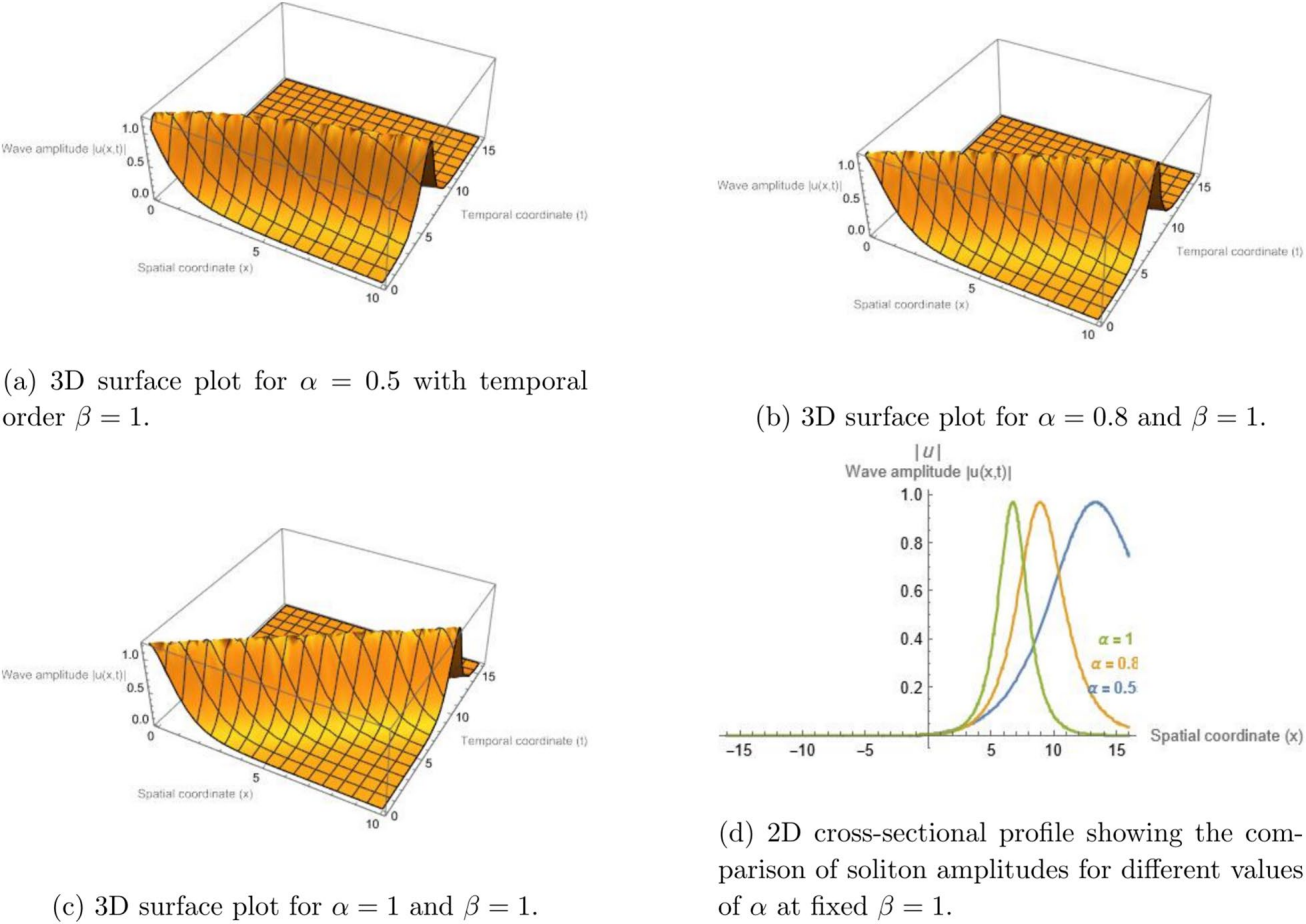
Bright soliton solution of Eq. (13) illustrating the influence of the spatial fractional order $\alpha$ while the temporal fractional order is fixed at $\beta = 1$. Panels (a–c) display three-dimensional surface plots of the wave amplitude |*u*(*x*, *t*)| for $\alpha = 0.5$, $\alpha = 0.8$, and $\alpha = 1$, respectively. In these plots, the horizontal axis represents the spatial coordinate *x*, the depth axis corresponds to the temporal coordinate *t*, and the vertical axis denotes the wave amplitude |*u*(*x*, *t*)|. Panel (d) presents a two-dimensional cross-sectional comparison of the corresponding soliton profiles for different values of $\alpha$. The results show that decreasing the spatial fractional order leads to broader soliton structures, reflecting the enhanced dispersive behavior associated with spatial fractional effects.

It should be noted that the graphical illustrations presented in this section correspond to the general space--time fractional formulation, where the spatial and temporal fractional orders $\alpha$ and $\beta$ are treated independently. This allows the separate influence of spatial fractional dispersion and temporal fractional-order effects on the wave dynamics to be examined. For completeness, the simplified case $\alpha = \beta$ may also be considered, in which the parametric analysis reduces to varying a single fractional parameter. In this situation, the qualitative behavior of the obtained solutions remains consistent with the trends illustrated in the present figures. Figure 1 illustrates the bright soliton solution given by Eq. (13) for the parameter set $\sigma = 5.75$, $\gamma = 2.5$, $\alpha = 1$, $h = 0.332$, and $d_{2} = 6.06$. The same parameters are employed in Fig. 2, except that $\beta = 1$ is fixed while different values of $\alpha$ are chosen to visualize the influence of the spatial fractional derivative on the resulting wave pattern.

**Fig. 3:**
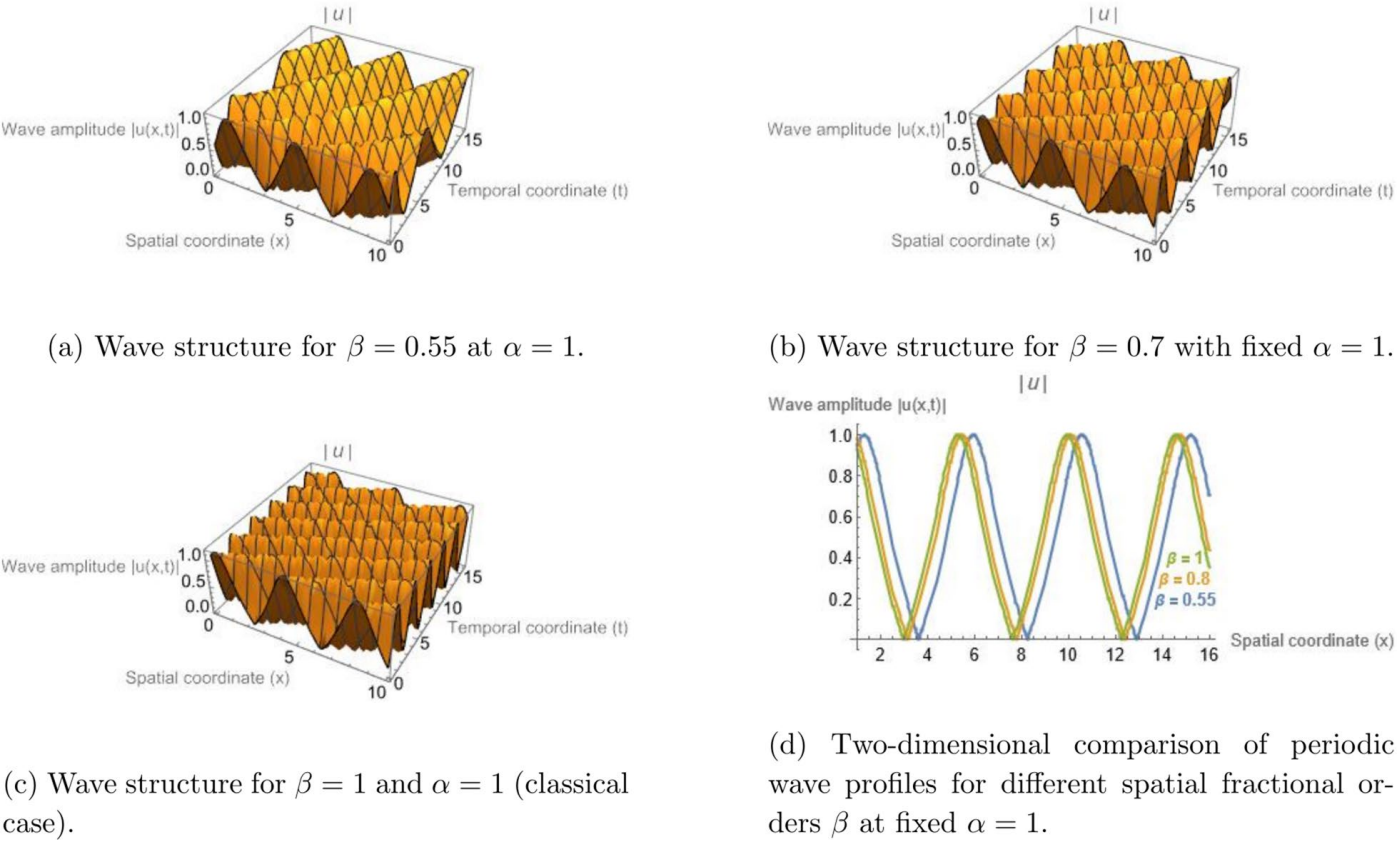
Jacobi elliptic cn-type periodic wave solution of Eq. (15) illustrating the influence of the temporal fractional order $\beta$ for a fixed spatial fractional order $\alpha = 1$. Panels (a–c) present three-dimensional surface plots of the wave amplitude |*u*(*x*, *t*)| for $\beta = 0.55$, $\beta = 0.7$, and $\beta = 1$, respectively. In these plots, the horizontal axis represents the spatial coordinate *x*, the depth axis corresponds to the temporal coordinate *t*, and the vertical axis denotes the wave amplitude |*u*(*x*, *t*)|. Panel (d) shows a two-dimensional cross-sectional comparison of the corresponding periodic wave profiles for different values of $\beta$. The results demonstrate how variations in the temporal fractional parameter influence the modulation and propagation characteristics of the periodic nonlinear wave.

**Fig. 4:**
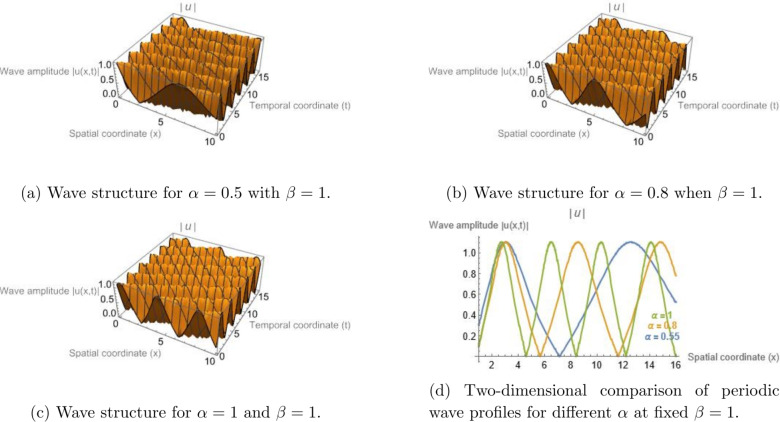
Jacobi elliptic cn-type periodic wave solution of Eq. (15) illustrating the influence of the spatial fractional order $\alpha$ while the temporal fractional order is fixed at $\beta = 1$. Panels (a–c) present three-dimensional surface plots of the wave amplitude |*u*(*x*, *t*)| for $\alpha = 0.5$, $\alpha = 0.8$, and $\alpha = 1$, respectively. In these plots, the horizontal axis represents the spatial coordinate *x*, the depth axis corresponds to the temporal coordinate *t*, and the vertical axis denotes the wave amplitude |*u*(*x*, *t*)|. Panel (d) shows a two-dimensional cross-sectional comparison of the corresponding periodic wave profiles for different values of $\alpha$. The results indicate that decreasing the spatial fractional order leads to wider spatial oscillations, reflecting the enhanced dispersive behavior introduced by spatial fractional effects.

**Fig. 5:**
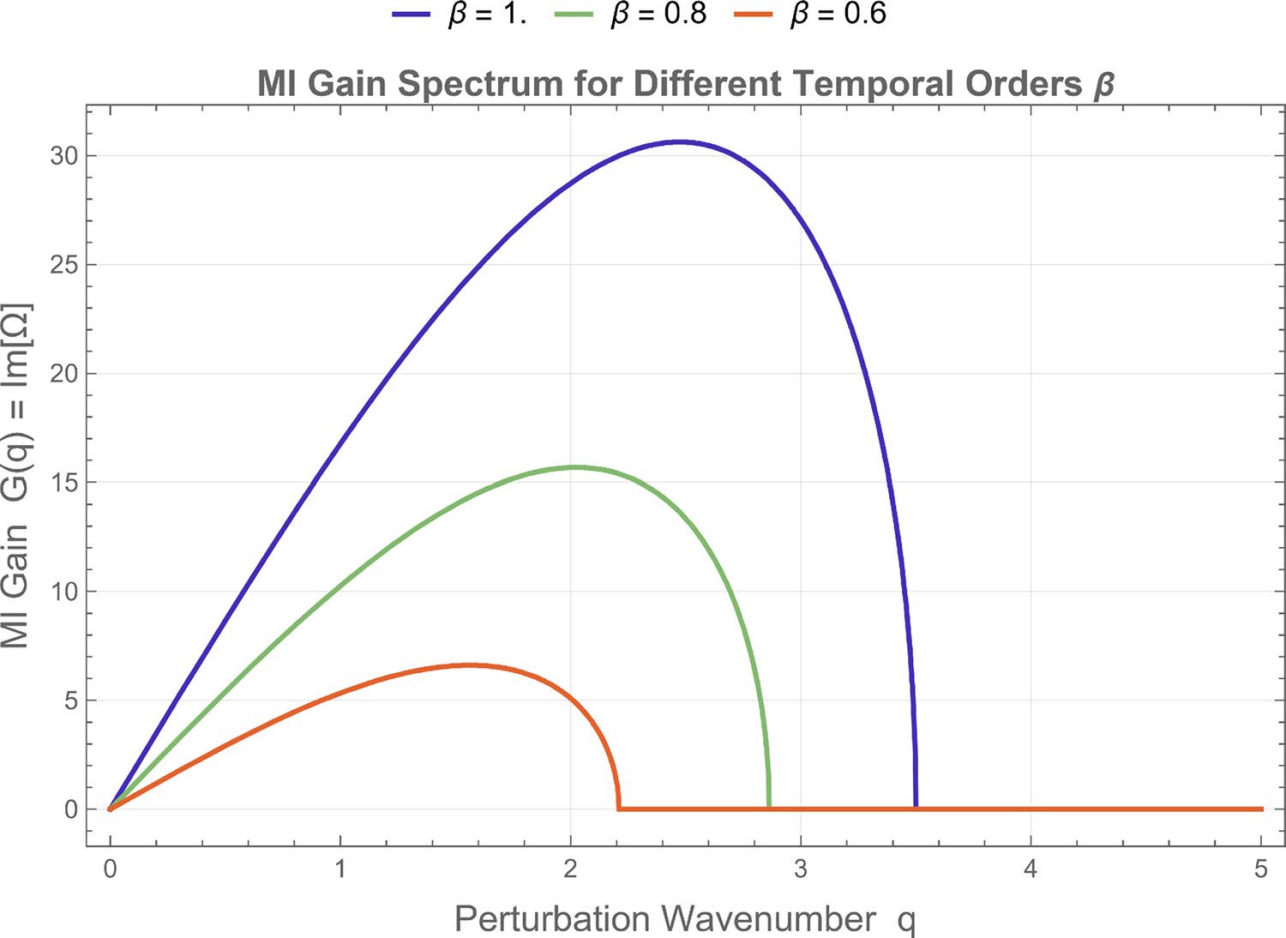
Modulation instability (MI) gain spectrum *G*(*q*) for different temporal fractional orders $\beta$ at fixed spatial fractional order $\alpha = 1$. The curves illustrate how decreasing $\beta$ reduces both the maximum instability gain and the width of the unstable spectral band. This behavior indicates that stronger temporal fractional effects suppress the growth of perturbations and enhance the stability of the continuous-wave background.

**Fig. 6:**
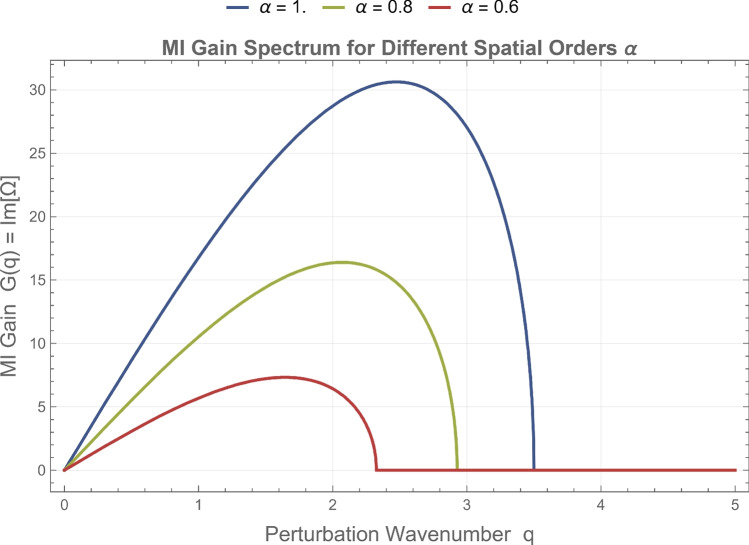
Modulation instability gain spectrum for various spatial fractional orders $\alpha$ with fixed temporal fractional order $\beta = 1$. The results show that decreasing $\alpha$ shifts the instability peak toward lower perturbation wavenumbers and slightly modifies the spectral distribution. This effect reflects the influence of spatial fractional dispersion on the stability characteristics of the nonlinear wave system.

Figure 3 displays the periodic Jacobi elliptic solution associated with Eq. (eq14) for $\sigma = -7$, $\gamma = 6.35$, $\alpha = 1$, $h = 0.382$, $d_{2} = 5$, and modulus $m = 0.952$. Correspondingly, Fig. 4 uses the same parameters but varies $\alpha$ (with $\beta =1$ held constant) to illustrate how the fractional-order effect modifies the spatial structure of the periodic wave. All graphical simulations were produced using Mathematica 13.3 with a resolution of $500 \times 500$ grid points for the parametric domain. The fractional parameters were selected to ensure physically consistent dispersion–nonlinearity balance, with $\alpha , \beta \in (0.6,1]$, representing typical optical and plasma media. The newly added Figs. 5 and 6 illustrate the modulation instability gain spectra for different fractional orders, providing quantitative insight into how fractional dispersion and temporal fractional-order influence soliton stability.

### Physical interpretation of fractional parameter effects

As observed in Figs. 1, 2, 3, and 4, varying the fractional parameters $\alpha$ and $\beta$ primarily causes a translation or phase shift of the solution profiles, while the amplitude of the solitons and periodic waves remains nearly unchanged. This behavior can be explained by analyzing the structure of the traveling-wave transformation and the properties of the employed fractional operators. The wave variable in Eq. ((8)) is defined as

$$\begin{aligned} z = h\!\left( \frac{1}{\Gamma (\alpha )} + x\right) ^{\!\alpha }\!/\alpha - \nu \!\left( \frac{1}{\Gamma (\beta )} + t\right) ^{\!\beta }\!/\beta , \end{aligned}$$

which acts as a fractional parameterizations of space and time. The parameters $\alpha$ and $\beta$ thus serve as scaling exponents that stretch or compress the coordinate axes. Consequently, variations in these parameters primarily result in horizontal displacements of the solution profile in the $(x,t)$ plane, rather than in changes to its peak amplitude. Mathematically, the amplitude of the derived solutions--e.g., the bright soliton

$$\begin{aligned} u(x,t) = h \sqrt{d_2} \, \textrm{sech}\!\big [\sqrt{d_2} \, \Xi (x,t)\big ] \exp \!\big [i\Phi (x,t)\big ], \end{aligned}$$

remains governed by the nonlinear coefficient balance between dispersion and self-phase modulation terms. Since the fractional orders $\alpha$ and $\beta$ appear in the argument $\Xi (x,t)$ but not in the amplitude perfector, they induce spatial-temporal shifts without altering the energy content of the pulse. From a physical standpoint, this indicates that the chosen $\beta$-fractional derivative preserves the normalization of the wave envelope, acting as a temporal scaling operator rather than as an amplitude-modulating mechanism. The invariance of amplitude with respect to fractional variation reflects the conservative nature of the underlying nonlinear Schrödinger-type dynamics.

In contrast, dissipative fractional derivatives (e.g., of Caputo–Fabrizio or Atangana–Baleanu type) would typically lead to amplitude attenuation or gain. Therefore, the observed invariance of soliton amplitude with fractional variation is intrinsic to the adopted $\beta$-derivative definition combined with the MEDM traveling-wave transformation, confirming that fractional parameters modify the phase and localization of the wave but not its energy-conserving amplitude.

### Quantitative influence of fractional parameters

A quantitative assessment of the fractional parameters reveals that decreasing the spatial order $\alpha$ broadens the soliton width and weakens localization, while lowering the temporal order $\beta$ primarily reduces the group velocity without significantly affecting amplitude. Analytical relations derived from the MEDM framework show that the soliton width $W \propto \alpha /[\Gamma (\alpha )\sqrt{d_2}]$ increases as $\alpha$ decreases, whereas the group velocity $v_g \propto (\Gamma (\alpha )/\Gamma (\beta ))(\beta /\alpha )$ decreases with smaller $\beta$. For the periodic Jacobi waves, the effective spatial period $\Lambda \propto \alpha K(m)/[\sqrt{d_2}\Gamma (\alpha )]$ also grows as $\alpha$ is reduced. Together, these quantitative relations confirm that the fractional orders act as tunable parameters that control the soliton’s width, propagation speed, and periodicity, thereby providing quantitative insight into how fractional dispersion modifies nonlinear wave localization.

## Linear stability analysis

We investigate the linear stability of the continuous-wave (CW) solution of the fractional extended NLS model (Eq. (eq1)). Our aim is to identify conditions under which small perturbations grow, indicating modulation instability (MI).

### Background state and perturbation ansatz

A uniform background solution is written as

$$\begin{aligned} u_0(x,t) = K\, e^{i\Phi _0(x,t)}, \qquad K=\text {const}, \end{aligned}$$

where $\Phi _0$ collects the background phase. We introduce a small perturbation,

$$\begin{aligned} u(x,t)=\bigl ( K + \tilde{\varepsilon }(x,t) \bigr )\,e^{i\Phi _0(x,t)}, \qquad |\tilde{\varepsilon }|\ll K, \end{aligned}$$

and expand the perturbation in the two-sideband form

$$\begin{aligned} \tilde{\varepsilon }(x,t) = a(t)e^{iqx} + b^*(t)e^{-iqx}, \end{aligned}$$

where *q* is the perturbation wavenumber. We assume harmonic time dependence $a(t), b(t) \sim e^{-i\Omega t}$, where $\Omega$ is the complex perturbation frequency.

### Linearization and reduced system

Substituting the perturbed field into Eq. (1) and keeping only linear terms in (*a*, *b*) yields a coupled $2\times 2$ algebraic system

$$\begin{aligned} \mathcal {M}(q,\Omega )\begin{pmatrix} a \\ b \end{pmatrix}=0. \end{aligned}$$

All contributions from dispersion, fractional derivatives, nonlinear dispersion, and cubic nonlinearity combine into three model-dependent coefficient functions *P*(*q*), *Q*(*q*), and *R*(*q*), which arise naturally from the linearization of:

- the linear dispersive operator (contributing to *P*),
- the self-phase modulation and mixed nonlinear terms (contributing to *Q*),
- the coupling between the two sidebands (giving *R*).

Their explicit expressions depend solely on the coefficients in Eq. (eq1) and can be computed directly from the model.

### Dispersion relation

Nontrivial solutions require $\det \mathcal {M}=0$, leading to the dispersion relation

18 $$\begin{aligned} \bigl (-\Lambda _t(\Omega )+P(q)+2K^2Q(q)\bigr ) \bigl (-\Lambda _t(\Omega )+P(-q)+2K^2Q(-q)\bigr ) - K^4 R(q)R(-q)=0 . \end{aligned}$$

For real model coefficients, $P(-q)=\overline{P(q)}$, $Q(-q)=\overline{Q(q)}$ and $R(-q)=\overline{R(q)}$, so the relation becomes a quadratic equation in $\Lambda _t(\Omega )$:

$$\begin{aligned} \Lambda _t(\Omega )^2 - 2\Lambda _t(\Omega )\Re \bigl (P(q)+2K^2Q(q)\bigr ) + \Delta (q,K) = 0, \end{aligned}$$

where

$$\begin{aligned} \Delta (q,K)=|P(q)+2K^2Q(q)|^2 - K^4|R(q)|^2 . \end{aligned}$$

Solving for $\Lambda _t(\Omega )$ yields

19 $$\begin{aligned} \Lambda _t(\Omega )= \Re \bigl (P(q)+2K^2Q(q)\bigr ) {\pm } \sqrt{K^4|R(q)|^2 - \Im \bigl (P(q)+2K^2Q(q)\bigr )^2 } . \end{aligned}$$

### Growth rate and modulation instability (MI)

The temporal growth rate follows from the fractional-time relation $\Lambda _t(\Omega )=(-i\Omega )^\beta$. Instability occurs when $\textrm{Im}(\Omega )>0$, which requires the discriminant

$$\begin{aligned} \mathcal {D}(q,K)= K^4|R(q)|^2 - \Im \bigl (P(q)+2K^2Q(q)\bigr )^2 \end{aligned}$$

to be positive. Thus MI arises whenever

$$\begin{aligned} \boxed {\;\mathcal {D}(q,K) > 0\;} \end{aligned}$$

and a branch of $(-i\Omega )^\beta$ is chosen that yields $\textrm{Im}(\Omega )>0$. For weak instabilities one may approximate

$$\begin{aligned} \Omega \approx i\bigl (\Lambda _t\bigr )^{1/\beta }, \end{aligned}$$

so that the MI gain is $\Gamma (q)=\textrm{Im}(\Omega )$.

### Classical limit: $\alpha =\beta =1$

When the model reduces to the classical NLS (integer derivatives and no higher-order terms), $\Lambda _t(\Omega )=-i\Omega$, and the dispersion relation becomes a quadratic equation for $\Omega$:

$$\begin{aligned} \Omega = i\,\Re \bigl (P(q)+2K^2Q(q)\bigr ) {\pm } i\sqrt{K^4|R(q)|^2 - \Im \bigl (P(q)+2K^2Q(q)\bigr )^2}. \end{aligned}$$

MI occurs whenever the quantity inside the square root is positive, reproducing the standard NLS modulation-instability criterion.

### Modulation instability gain and fractional effects

To visualize the impact of fractional parameters on stability, the modulation instability (MI) gain spectrum is evaluated using $G(q) = \operatorname {Im}[\Omega (q)],$ where $(\Omega (q))$ is determined from the dispersion relation in Eq. (eq19). The corresponding MI gain curves are presented in Figs. 5 and 6, illustrating how the fractional orders $\alpha$ and $\beta$ govern the instability bandwidth and peak amplitude. Figure 5 displays the MI gain spectra for different temporal fractional orders $\beta$ at a fixed spatial order $\alpha =1$. As the temporal order decreases ($0.7 \le \beta < 1$), both the maximum gain and the width of the instability band are reduced, signifying that lower $\beta$ values suppress modulation instability and enhance pulse stability. Figure 6 shows the influence of the spatial fractional order $\alpha$ at a fixed temporal order $\beta =1$; decreasing $\alpha$ shifts the instability region toward lower wavenumbers and slightly broadens the spectral base, reflecting the increased nonlocal dispersive effect introduced by the fractional derivative. Overall, the fractional parameters act as stability–control factors: lower $\beta$ values damp the temporal growth of perturbations, whereas smaller $\alpha$ values redistribute and smooth the unstable spectrum in $q$-space. These effects confirm that the fractional NLS framework provides tunable management of stability regions, offering potential applications in optical and plasma systems where the dispersion–nonlinearity balance must be dynamically regulated.

## Conclusion

The findings of this study underscore the critical importance of incorporating fractional derivatives when analyzing soliton propagation. The results vividly demonstrate how the order of the fractional derivative significantly influences the behavior of the soliton signal. Specifically, the fractional derivatives introduce additional degrees of freedom in the model, allowing for a more precise description of the wave dynamics in complex media. A detailed linear stability analysis was performed to examine the modulation behavior of continuous-wave backgrounds within the fractional extended NLS framework.

The results presented in this study reveal clear physical trends governed by the fractional parameters $\alpha$ and $\beta$. A decrease in the spatial fractional order $\alpha$ enhances the degree of dispersion, leading to broader and less localized soliton profiles. Conversely, a reduction in the temporal fractional order $\beta$ modifies the temporal scaling of the wave evolution, which can suppress modulation instability and promote more stable propagation. These findings demonstrate that fractional derivatives provide a versatile framework for tuning the balance between nonlinearity and dispersion. The fractional parameters thus serve as control variables that regulate soliton width, amplitude, and energy localization. This deeper interpretation highlights how fractional calculus not only generalizes classical soliton models but also establishes new pathways for the design and control of nonlinear wave phenomena in real-world physical media. Beyond the mathematical and numerical analysis, the present results have important implications for several applied fields in which nonlinear wave propagation governs the underlying physical dynamics. The fractional nonlinear Schrödinger-type model considered here provides a generalized framework applicable to nonlinear optics, plasma dynamics, metamaterials, and fluid systems. In nonlinear optical fibers and photonic crystals, fractional parameters can be engineered through spatially varying dispersion or gain–loss profiles, enabling tunable control of pulse width and stability. In plasma environments, the model captures the influence of non-Maxwellian distributions and long-range particle correlations on wave localization. Similarly, In fluid dynamics, similar nonlinear Schrödinger-type frameworks describe the evolution of internal solitary waves in stratified oceans, capillary–gravity waves on shallow surfaces, and wave packets in viscoelastic or turbulent fluids. The inclusion of the fractional orders $\alpha$ and $\beta$ provides a powerful means of incorporating hereditary response, and anomalous diffusion effects, which are characteristic of real-world fluid environments. The results indicate that fractional parameters can be tuned to control soliton width, stability, and energy localization, offering potential applications in the mitigation of rogue wave formation, the design of energy-efficient fluid channels, and the modeling of coastal or atmospheric wave dynamics. These insights extend the practical applicability of fractional nonlinear models beyond optics and plasma physics to broader fluid dynamical systems governed by nonlocal interactions. These applications demonstrate that the fractional framework is not only of theoretical interest but also offers a flexible foundation for designing and controlling nonlinear wave phenomena in real-world physical systems.

## Data Availability

The datasets used and/or analysed during the current study are available from the corresponding author on reasonable request
